# Protocols and Programs for High-Throughput Growth and Aging Phenotyping in Yeast

**DOI:** 10.1371/journal.pone.0119807

**Published:** 2015-03-30

**Authors:** Paul P. Jung, Nils Christian, Daniel P. Kay, Alexander Skupin, Carole L. Linster

**Affiliations:** 1 Luxembourg Centre for Systems Biomedicine, University of Luxembourg, Esch-sur-Alzette, Luxembourg; 2 National Center for Microscopy and Imaging Research, University of California San Diego, La Jolla, California, United States of America; University of Strasbourg, FRANCE

## Abstract

In microorganisms, and more particularly in yeasts, a standard phenotyping approach consists in the analysis of fitness by growth rate determination in different conditions. One growth assay that combines high throughput with high resolution involves the generation of growth curves from 96-well plate microcultivations in thermostated and shaking plate readers. To push the throughput of this method to the next level, we have adapted it in this study to the use of 384-well plates. The values of the extracted growth parameters (lag time, doubling time and yield of biomass) correlated well between experiments carried out in 384-well plates as compared to 96-well plates or batch cultures, validating the higher-throughput approach for phenotypic screens. The method is not restricted to the use of the budding yeast *Saccharomyces cerevisiae*, as shown by consistent results for other species selected from the Hemiascomycete class. Furthermore, we used the 384-well plate microcultivations to develop and validate a higher-throughput assay for yeast Chronological Life Span (CLS), a parameter that is still commonly determined by a cumbersome method based on counting “Colony Forming Units”. To accelerate analysis of the large datasets generated by the described growth and aging assays, we developed the freely available software tools GATHODE and CATHODE. These tools allow for semi-automatic determination of growth parameters and CLS behavior from typical plate reader output files. The described protocols and programs will increase the time- and cost-efficiency of a number of yeast-based systems genetics experiments as well as various types of screens.

## Introduction

A major goal in modern biological research is to determine the genetic basis of phenotypic variability. Different approaches can be employed to reach this aim. One approach consists in comparing a large number of case and control samples of an entire population in so-called genome-wide association studies (GWAS). Alternatively, linkage mapping surveys that investigate progeny from phenotypically divergent parents can be applied. Yeasts, and especially the budding yeast *Saccharomyces cerevisiae*, are attractive model organisms for genetic linkage studies given their fast growth, the high density of genetic markers, the possibility of generating relatively easily large haploid segregant collections, and the conservation of many biological processes between yeasts and higher eukaryotes. The linkage approach has been used intensively to determine the genetic basis of a number of phenotypic traits in yeast including thermotolerance, ethanol tolerance, and ammonium toxicity resistance [1–4].

Since growth is a main microbial objective, a classical yeast phenotyping method is to estimate the cell number in batch cultures with distinct liquid media over time by measuring the optical density at 600 nm (defined as OD throughout the article). Other commonly used phenotypic readouts are colony size and morphology after cultivation on solid media [5–7]. Significant advantages of growth monitoring in liquid culture versus colony size estimation include the ability of detecting more subtle growth kinetic variations and of determining more parameters per sample (lag time, growth rate, and yield of biomass versus colony size only).

As yeast-based systems biology approaches (genome-wide chemical or genetic screens, Quantitative Trait Locus analyses) require high-throughput phenotypic assays, automated growth assays based on liquid microcultivations in 96- to 100-well plates in thermostated and shaking plate readers have been developed and are widely used [8–11]. However, the 384-well plate format is typically not exploited for such assays and corresponding informatic tools to extract quantitative fitness parameters from growth curves remain either rather specific or inconvenient and often not easily accessible. Toussaint and colleagues [12] described an algorithm to calculate growth rates and lag times (Perl script available upon request). More user-friendly approaches like a macro for Excel files or R packages are available on demand or online [13, 14]. Very recently, a freely available software (GrowthRates) for treating growth datasets obtained from bacterial microcultivations has been published [15]. Our here developed open source bioinformatic tool combines a user-friendly graphical interface with underlying flexible Python classes that can be easily adapted to other potentially desired analysis pipelines.

Although yeast aging is a specific phenotype that remains both difficult and time-consuming to determine, linkage mapping studies have recently associated Quantitative Trait Loci (QTLs) with replicative aging in *S*. *cerevisiae* [16, 17]. To the best of our knowledge, no such studies have so far been implemented to explore the genetic basis of yeast chronological aging. Replicative lifespan (RLS) in yeast is defined as the number of daughter cells that a single mother cell produces before entering cellular senescence where cells do not divide any further [18]. In contrast, chronological life span (CLS) is defined as the time cells can survive once they entered senescence [18, 19]. Both aging features have been investigated in *S*. *cerevisiae* mostly using strains from gene deletion collections. These studies have played a pivotal role in the discovery of widely conserved signalling pathways involved in the regulation of life span from yeast to humans [20–25].

One bottleneck in existing yeast RLS and CLS assays is the number of conditions and/or strains that can be studied in parallel in the same experiment for comprehensive analyses. Given the inherent lengthy nature of aging experiments, it is obvious that increasing this number is a critical aim to reach for implementing throughput-intensive experiments. The classical approach for RLS determination consists in removing and counting daughter cells from larger mother cells through microdissection [26]. Recently, more high-throughput and automated strategies have been developed using microfluidic platforms where mother cells are trapped in specific chambers and the RLS is determined by the number of daughter cells produced or the number of bud scars on the mother cell [27–29]. None of these microfluidic setups is commercially available and, as they cannot be easily recreated, the vast majority of laboratories continue to depend on the classical, low-throughput approach [18]. To measure the CLS, the classical approach consists in repeatedly plating cells over time from aging cultures onto solid media plates and counting Colony Forming Units (CFU) [30]. It has recently been established that measuring the outgrowth kinetics of aging cultures by monitoring OD over time is also suitable for CLS determination [31]. The method relies on measuring the progressive increase in lag time for outgrowth curves generated from the same aging culture on successive days. This increase is caused by a decrease in the number of viable cells in the aging culture. Until now, the outgrowth method has been used within experimental setups using a non-standard 100-well plate format for CLS assays [31, 32].

Here, we have developed higher-throughput methods for yeast growth phenotyping and CLS determination based on microcultivations in standard 384-well plates. We complement these methods with the newly developed semi-automated open source software tools GATHODE (Growth Analysis Tool for High-throughput Optical Density Experiments) and CATHODE (Chronological life span Analysis Tool for High-throughput Optical Density Experiments) to analyse the data generated by our growth and aging assays. The semi-automation of GATHODE is highly useful to avoid errors in high-throughput determinations of fitness parameters. The described experimental protocols and software tools are expected to substantially increase both the time- and cost-efficiency as well as the accuracy of systematic yeast-based high-throughput studies involving fitness or life span measurements.

## Materials and Methods

### Species and Media

The *S*. *cerevisiae* strains that were used in this study are listed in S1 Table [33]. Other species used were: *Saccharomyces exiguus* (CBS379), *Zygosaccharomyces rouxii* (CBS732), *Kluyveromyces lactis* (CLIB210), *Saccharomyces* (*Lachancea*) *kluyveri* (CBS3082), *Pichia* (*Millerozyma*) *sorbitophila* (CBS7064), *Pichia* (*Millerozyma*) *farinosa* (CBS185), and *Yarrowia lipolytica* (CLIB89). Cells were grown in YPD medium (2% peptone, 1% yeast extract, 2% glucose) for setting up the microplate growth assay and different concentrations of NaCl were added for preliminary phenotypic analyses. Trait profiling analysis was performed in minimal YNB medium (defined throughout this study by 6.7 g/l Yeast Nitrogen Base with ammonium sulfate from MP Biomedicals supplemented with 2% glucose) supplemented with various chemicals (S2 Table). To test for the ability to grow with alternative carbon sources, glucose was replaced by galactose or maltose in two of the conditions tested (S2 Table). Aging experiments were performed in YNB medium, synthetic complete medium (6.7 g/l Yeast Nitrogen Base, 2 g/l SC Amino Acid mixture from MP Biomedicals) with 2% glucose (SC) or synthetic complete medium with 0.5% glucose for caloric restriction (CR). The detailed composition of the synthetic complete medium is given in S3 Table.

### Growth Phenotyping Assay

We adapted the growth phenotyping assay described by Toussaint and Conconi [8] to the use of 384-well microplates. Briefly, single colonies were grown overnight in tubes or in 96-well microplates in biological duplicates. 2 μl aliquots of the overnight cultures were inoculated into a final volume of 80 μl in 384-well microplates (initial OD ≈ 0.005–0.02). Specific wells were inoculated with medium only for background correction purposes. These plates were then shaken in a thermostated microplate reader (TECAN M200 Infinite Pro) at 30°C for 280 cycles of orbital (8 min at 140 rpm) and linear (2 min at 300 rpm) shaking followed by OD measurements (about 48 hours in total). A ‘Sample_Condition’ label was assigned to each well by the Magellan software (TECAN) where ‘Sample’ describes the strain or species and ‘Condition’ the medium in the corresponding well. This labelling allowed for coherent background correction using the GATHODE software described below. Using the plate reader software, the raw OD data were exported as tables in ASCII format with the addition of kinetic time stamps and temperature values.

### OD Correction at High Cell Density

The high accuracy of the OD values obtained during batch cultures is based on appropriate dilution of an aliquot of the culture prior to the OD measurement. To overcome the low saturation limit of optical detectors in microplate readers, it has been shown that, for automated bacterial and yeast microcultivation experiments, one can correct for the non-linearity of OD measurements in high-density cultures by comparing measured ODs with ODs calculated based on the dilution factor [13, 34]. Accordingly, we concentrated flask cultures that had reached OD ≈ 0.6 (measured in a standard 1-cm cuvette in a conventional spectrophotometer) ten-fold and took OD measurements of serial dilutions of the concentrated cultures (starting OD ≈ 6). Plotting the calculated OD values (based on the dilution factor) against the measured OD values (microplate reader), we observed a clear functional relationship between both datasets (S1A Fig.). This relationship seems to be more generally applicable as the values obtained with different *S*. *cerevisiae* strains can be fitted by the same polynomial function (S1A Fig.). Correction of the OD values collected during our yeast microcultivations with the polynomial function allowed for a more accurate determination of specific growth rates and a more nuanced comparison of the yield of biomass produced by different strains or in different conditions (S1B and S1C Figs.).

### Calculation of Growth Parameters

For each well, the optical density OD was measured at specific time points *t∈*[0;n] (note that *t* is the index of data arrays; whenever we refer to the real time we explicitly write *T(t)*). Growth parameters were calculated for a single well and subsequently the arithmetic mean and variance were determined from multiple wells corresponding to the replicates. We denote the set of wells for a specific ‘Sample_Condition’-tuple by *W* (sample, condition). Using the wells filled with medium only, the arithmetic mean of the raw readout of their optical density for each point in time, denoted by ${\hat{OD}}_{t}^{blank,condition}$, was used as a background reference:

$${\hat{OD}}_{t}^{blank,condition}=\frac{\sum_{i\in W(blank,condition)}{\hat{OD}}_{t}^{i}}{\left|W(blank,condition)\right|}$$

where the denominator denotes the number of wells filled with medium only.

For each well *i*, the background-corrected optical density is given by:

$$OD_{t}^{i,condition}={\hat{OD}}_{t}^{i,condition}-{\hat{OD}}_{t}^{blank,condition}$$

In other words, at each time point, the average background OD is calculated for a given medium and subtracted from every sample replicate (medium plus cells).

To extract the maximal growth rate for each cultivation, a fit of an exponential function to the data was required. Since the data did not exhibit an exponential form on a large scale, the fit was performed piecewise for small intervals of ω data points:

$$(\mu_{t+\frac{\omega }{2}},OD_{t+\frac{\omega }{2}}^{\left(0\right)})=\underset{\mu ,OD^{\left(0\right)}}{\arg\;\min}\overset{t+\omega }{\underset{t'=t}{\sum }}{\left(OD^{\left(0\right)}e^{\mu T(t')}-OD_{t'}\right)}^{2}$$

The maximal growth rate (*μ*_*max*_) is the maximal *μ* determined this way, and the corresponding lag time (*t*_*lag*_) is calculated as the intersection of the exponential function with a given OD value. In our study, inoculation volumes were the same for all the tested wells in a given experiment, leading to virtually identical starting ODs, which were then chosen as the baseline for lag time calculation. The yield of biomass is defined as the OD reached in stationary phase. This growth parameter was extracted by performing a linear regression in a given time window and taking the maximal OD of all data points that led to a slope compatible with zero.

### Chronological Life Span Assay

Single colonies were grown in 5 ml (tubes) or in 200 μl (96-well microplates) YPD medium overnight at 30°C in 3 biological replicates. 50 μl of these pre-cultures were used to inoculate 1 ml fresh medium in 96-deep-well plates (Greiner Bio-One) or 15 ml fresh medium in 125 ml-flasks. Flasks were shaken at 200 rpm whereas 96-deep-well plates were shaken at 1000 rpm to avoid sedimentation of the cells. For the 96-deep-well plates, a thermostated incubator (Edmund Bühler GmbH) allowing simultaneous high-speed shaking of a maximum of four plates was used. The plates were sealed with adherent foil (AeraSeal) and a beaker filled with water was added into the incubator to minimize medium evaporation. Aliquots (2 μl) of the aging cultures were sampled every 2–3 days during 2 weeks and inoculated into 78 μl YPD medium in a 384-well microplate. The latter was then subjected to the same kinetic cycle with OD monitoring in a TECAN M200 Infinite Pro plate reader as described for the growth phenotyping assay. Doubling times and lag times were extracted from the generated growth curves and used to determine the proportion of living cells in the aging cultures. As described by Murakami and colleagues [31], the survival percentage for each strain at each age-point relative to a defined initial time-point can be calculated according to the formula:

$$S_{n}=100\times \frac{1}{2^{\frac{\Delta t_{n}}{\delta }}}$$

where *S*_n_ is the survival percentage at age-point n, Δt_n_ is the time shift between the growth curves at the initial time-point and age-point n, and δ is the mean of the doubling times calculated for the strain at each individual age-point. The viability is defined to be 100% at the initial age-point (day 2 or 48 hours after inoculation of the main cultures). The survival integral (SI), namely the area under the survival curve, can be obtained by the formula:

$$SI=\sum_{2}^{n}\left(\frac{S_{n}+S_{n-1}}{2})(Age_{n}-Age_{n-1}\right)$$

where Age_n_ is the age-point (e.g. 2, 5, 7, 10, 12, 14 days) and *S*_n_ the viability at that age-point. The SI is commonly used as a means to quantify the CLS in aging studies and we use the terms SI and CLS interchangeably in this article.

## Results and Discussion

### High-throughput Kinetic Growth Assay

**Fig 1:**
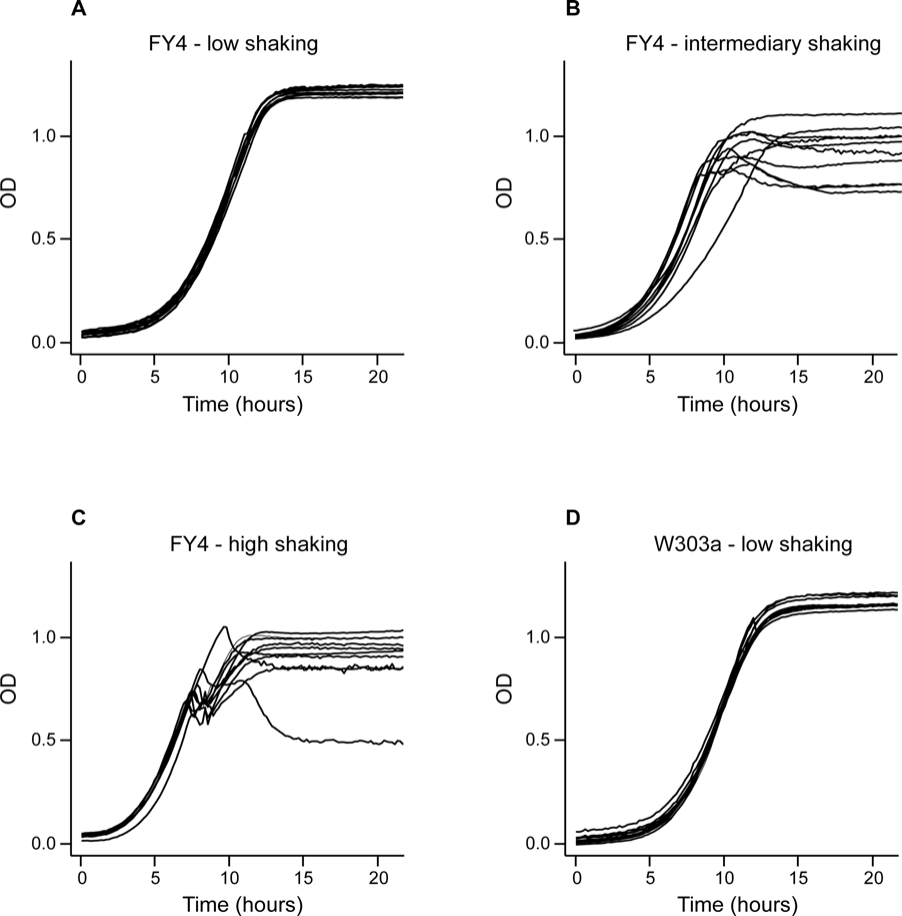
Optimization of the shaking parameters for yeast microcultivation in 384-well plates. Ten technical replicates of the *S*. *cerevisiae* FY4 strain (haploid state) in liquid YPD medium were subjected to shaking at lower (A), intermediary (B) and higher (C) speed with OD monitoring in a microplate reader at 30°C. Between each OD measurement, kinetic cycles consisted of 8 minutes of orbital shaking (140 rpm, 245 rpm and 430 rpm for low, intermediate and high shaking, respectively) followed by 2 minutes of linear shaking (300 rpm, 410 rpm and 890 rpm, respectively). Comparable growth curves were observed, but a better reproducibility was obtained for the lower shaking speeds. This high reproducibility with the optimized shaking parameters (low speed) was confirmed for the W303a strain (D). The curves shown were corrected for background absorbance in ‘medium-only’ wells but not for non-linearity of OD measurements at high cell density.

To develop growth kinetics-based phenotyping and CLS assays for throughput-intensive experiments, a first requirement was to scale up currently used microcultivation growth assays from 96- or 100-well formats to a 384-well format. During microcultivation experiments, yeast cells tend to aggregate and sediment on the bottom of the wells thereby altering OD recordings as a function of the shaking mode [11, 13]. To avoid this problem in the 384-well plates, we tested a range of different shaking movements, shaking speeds, and culture volumes (40–90μl). High shaking speed has been highly recommended for microcultivation in 100-well plates [11, 13]. In our experimental setting, however, the best reproducibility in the 384-well format was obtained with a culture volume of 80 μl per well and relatively low shaking speeds using cycles that combine 8 minutes of orbital shaking (140 rpm) and 2 minutes of linear shaking (300 rpm) between consecutive OD measurements. Under these conditions, virtually identical growth curves were obtained for 10 technical replicates where OD was monitored in 10 different wells filled with culture aliquots derived from the same starting culture (Fig. 1A). This may be explained by the shape of the wells in 384-well microplates (square as opposed to round in the 100-well plates) resulting in a better homogenization of the cell suspensions even at lower shaking speed [35]. In contrast, higher shaking speeds resulted in clear discrepancies between growth curve replicates particularly in the saturating high density phase (Figs. 1B and 1C).

In the optimized conditions, highly reproducible growth curves were obtained for the lab *S*. *cerevisiae* FY4 strain (a derivative of the widely used S288c reference strain) in both the haploid state (Fig. 1A) and diploid state (not shown) as well as for the W303a strain (Fig. 1D). This consistency is further emphasized by similar results obtained with other yeast species, including *S*. *kluyveri* and *K*. *lactis* (S2 Fig.), suggesting that the reproducibility is not restricted to one specific strain or species. However, we were unable to obtain interpretable growth data for the yeast *Y*. *lipolytica*, likely caused by its dimorphic transition [36]. Overall, these data indicate that our growth assay protocol can be used with *S*. *cerevisiae* as well as with a number of other yeast species, to reproducibly determine the lag phase, the specific growth rate, and the yield of biomass.

To investigate respiratory growth, strains were cultivated in 96- or 384-well plates in the presence of non-fermentable carbon sources such as glycerol and ethanol. Under these conditions, only slow, quasi-linear growth was observed. Independently of the microplate format, we were also unable to detect a diauxic shift during growth in the presence of different glucose concentrations (0.2%, 0.5% and 2%) (S3 Fig.). In agreement with a previous study using a different microplate reader (BioScreen C) and higher shaking speeds [13], this indicates that yeast microcultivations are not generally suitable for respiratory growth studies, possibly due to limitations in oxygen supply under these assay conditions.

### Growth Analysis Tool (GATHODE)

**Fig 2:**
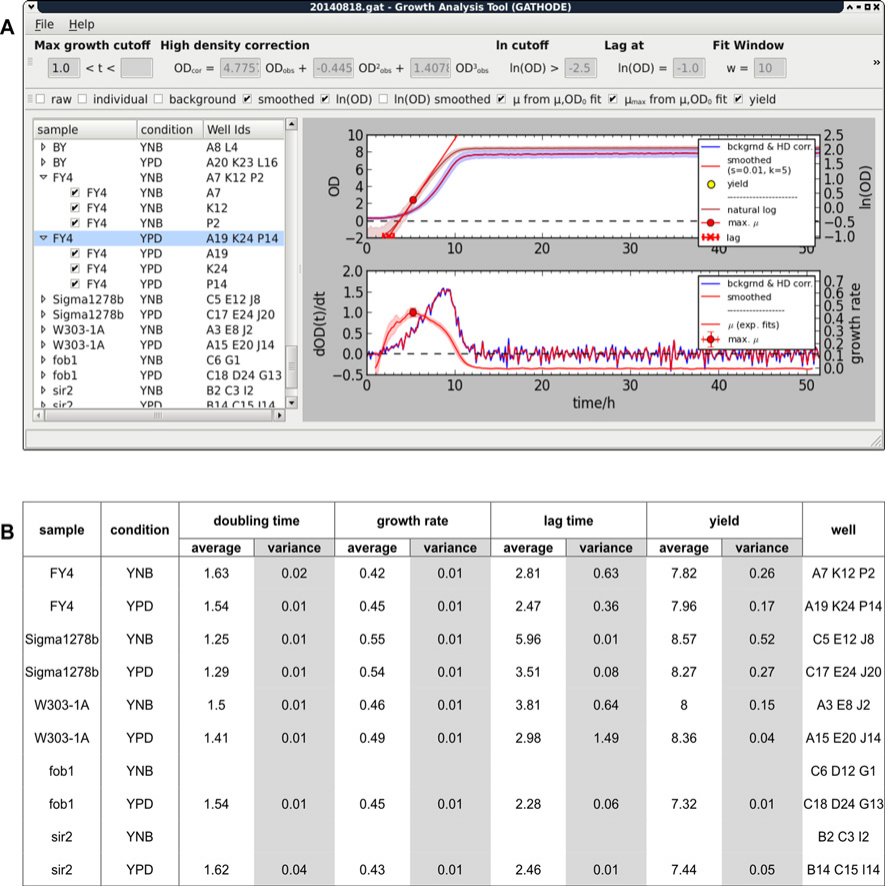
Growth Analysis Tool (GATHODE) software. A) Screenshot of the GATHODE software depicting, in the top graph, average growth curves (generated from corrected non-transformed and log-transformed OD measurements) for 3 biological replicates (FY4 strain grown in YPD). The shading around the growth curves represents the standard deviation. The derivative of OD with respect to time is represented in the bottom graph and the maximum of this curve corresponds to the maximal growth rate. A list with the tested strains and corresponding growth media and well IDs is automatically generated based on sample labelling in the left part of the window. The settings of a number of parameters (e.g. OD correction for high-density cultures, curve smoothing, time window within which to determine growth rate, baseline OD for lag time determination) can be changed in the upper part of the window. Extensive help documentation is made available with the software package for more detailed information. B) The extracted growth features can be exported for further processing to a. csv file either as averages (with corresponding variances) of replicates as shown in this table or as single well values.

To extract the relevant growth parameters from such 384-well plate microcultures in a semi-automated way, we developed, in close collaboration between experimentalists and theoreticians, a computer program for analysis of the resulting growth curves (GATHODE). The program consists of the main analysis logic suitable for batch processing and a graphical user interface (GUI) that, unlike the recently described GrowthRates software [15], allows to quickly inspect growth curves, adjust extraction parameters, blacklist outliers, and generate figures (Fig. 2A). Independent of the microplate format, GATHODE will determine 3 growth parameters: the maximal growth rate as the slope of the logarithmic transformed OD data represented as a function of time (closely related to the doubling time), the lag time as the interception of this slope with the baseline, and the yield of biomass. The extracted growth parameters can be exported to a. csv file for further analyses and/or generation of figures. The user can choose to export the individual results obtained for each measured well or averages and variances of the values obtained for replicate wells (Fig. 2B).

In a typical experimental design, several strains and conditions will be analysed in different wells of one plate, including control wells containing only medium for background correction (see Materials and Methods). The resulting OD time series will be exported using the plate reader software in ASCII format with a unique labelling scheme containing the strain’s name and the condition in which it was grown, separated by an underscore (e.g. “FY4_YPD”). The exported file is then opened in the GUI that automatically groups growth curve replicates from the same strain and condition based on the labels (see Fig. 2A).

Default values for most of the analysis extraction parameters are pre-defined in GATHODE, including curve smoothing parameters, an OD threshold below which specific growth rates should not be determined, the interval size for exponential fitting, a time interval within which the specific growth rate should fall and the baseline OD value at which lag times are calculated (Fig. 2A). The high-density correction should, however, be determined in each specific laboratory setting based on the strategy described in Materials and Methods, as it may vary according to the type of plate reader used and the size of the analysed microbial cells. More documentation about parameters is made available with the software package.

In a first processing step, the average background OD measured in ‘medium-only’ control wells is automatically subtracted from the probe wells. From the background-corrected growth curves, the maximal growth rates and lag times are calculated under the assumption of exponential growth for each individual well, and the automated grouping ensures the accurate determination of strain- and condition-dependent averages and variances.

Given the unavoidable variability of even such standardized experiments, the software applies sanity checks and allows the user to adjust parameters of the underlying extraction algorithms to optimize the detection of the required quantities. For example, when carrying out local fits of an exponential function to the data to extract the maximal growth rate (see Materials and Methods), the program requires certain criteria to be fulfilled:

- the maximum growth rate (μ_max_) must be positive
- the initial OD must be positive
- the OD at the time of the maximal growth rate must not be below a given threshold (at low OD values the relative level of noise increases, which may lead to erroneous growth rate determinations).
- the time of the maximal growth rate should fall into a user-supplied interval, and it must not be at the endpoints of the interval (as this would mean that a local maximum could not be found). This strict requirement of a local maximum can be loosened by setting the parameter “*allow at cutoff*”, which mathematically means that the derivative of the growth rate may be non-zero. If this is the case, a red warning will appear in the GUI.

This mixed heuristic- and manual intervention-based approach enables a fast and robust evaluation of growth curves. The final growth analysis data together with the parameter settings can be saved as a. gat file that can be reloaded by GATHODE, allowing for the persistence and reproducibility of the results. The generated. gat files can also be loaded by the CLS Analysis Tool software (CATHODE) to determine chrolonological life span (see below).

The GATHODE and CATHODE tools are free and open source and are implemented in the Python programming language using the comprehensive numerical libraries numpy and scipy [37] (http://www.scipy.org/). The visualization is based on the plotting system matplotlib [38] and the graphical user interface is implemented with the PyQt framework (http://www.riverbankcomputing.co.uk/software/pyqt). The open source strategy makes the purchase of expensive proprietary mathematical software unnecessary and allows for an easy and cheap deployment of the program on an unlimited number of computers. Furthermore, the strict separation of analysis logic and graphical user interface ensures that the analysis can easily be used as a module in other custom workflows, which is also encouraged by the open source license strategy and the well-documented code. The software projects and their documentation are available at https://platereader.github.io/. The programs and their dependencies can easily be downloaded and installed on all major operating systems such as Linux, Mac OS and Microsoft Windows, without the requirement to set up a webserver and a database, as opposed to the software developed by Olsen and colleagues [39]. The use of the high-level programming language Python leverages the obstacle for less experienced developers to adapt and modify parts of this implementation for their customized projects.

### Application of the Growth Assay to Trait Profiling

**Fig 3:**
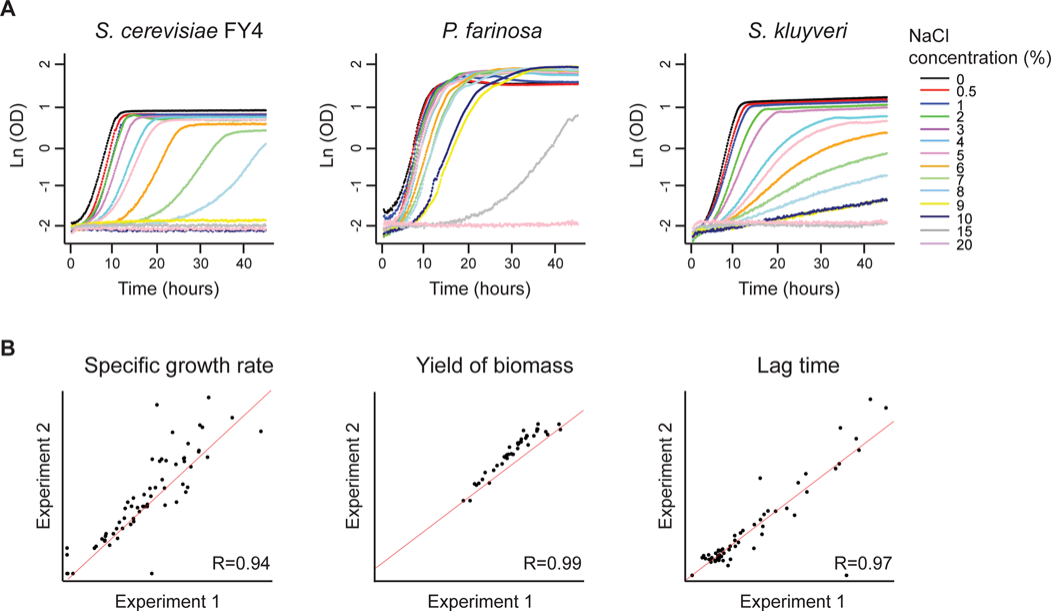
Application of the microcultivation growth assay to the analysis of salt resistance in several yeast species and inter-experimental reproducibility of this assay. A) Growth was measured in a microplate reader using liquid cultures supplemented with increasing NaCl concentrations (w/v) in 384-well plates for *S*. *cerevisiae* (FY4 strain), *P*. *farinosa* and *S*. *kluyveri*. For the sake of clarity, the curves shown were not corrected for background absorbance or non-linearity of OD measurements at high cell density. B) Growth was measured using this same assay for various *S*. *cerevisiae* strains (FY and W303 strains in both haploid and diploid states) and other yeast species (*K*. *lactis*, *P*. *farinosa*, *P*. *sorbitophila*, *S*. *kluyveri*, *Z*. *rouxii*) cultivated in the presence of different NaCl concentrations in two identical, but independent experiments. Growth parameters were determined using the GATHODE software. The values shown are means of three biological replicates.

As mentioned previously, growth in different conditions, and more specifically the specific growth rate, is a commonly used parameter for trait profiling in yeast [9–11]. To verify the applicability of our growth assay in 384-well microplates to yeast phenotyping, we cultivated *S*. *cerevisiae* strains as well as other yeast species in the presence of different NaCl concentrations. All tested *S*. *cerevisiae* strains showed growth in media containing up to 8% NaCl, with a gradual decrease of the growth rates and the yields of biomass, and a shift in lag time with increasing salt concentration (Fig. 3A). Similar results were previously obtained with microcultivations in 96-well plates [13]. Consistent with its description as an osmotolerant yeast species [40], *P*. *farinosa* showed growth up to 15% NaCl (Fig. 3A), the highest resistance found among the species tested in this study. The variability of the stationary phase OD values obtained for different NaCl concentrations with *P*. *farinosa* is due to a pseudo-biofilm that developed on top of the cultures; this was observed with no other species tested except for the closely related species *P*. *sorbitophila* [41, 42]. Interestingly, *S*. *kluyveri* was the second most NaCl-resistant species among the ones tested here, showing growth in media containing up to 10% NaCl (Fig. 3A). Taken together, these results indicate that our microcultivation method reproduces species-specific traits previously described by others [13, 40] and allows for detecting even subtle phenotypic variations due to environmental changes or between different yeast species.

To verify the reproducibility of our method, two independent, but identical experiments were carried out, involving cultivations of various yeast strains and species in the presence of different salt concentrations. Highly correlated values were obtained, using the GATHODE software, for all growth parameters (Fig. 3B), even for the lag time, which was previously reported as a less reproducible parameter in yeast microcultivation experiments [13]. Good correlations were also found when comparing growth of six strains in the presence of four different NaCl concentrations using 384- or 96-well plate microcultures (Pearson’s correlation coefficients, R, were 0.83, 0.88, and 0.74 for growth rate, biomass production, and lag time, respectively; S4A-C Figs.). Finally, the growth rates obtained in 384- or 96-well microplates and those obtained in batch cultures for the same strains cultivated in the same media were also highly correlated (R = 0.90 and R = 0.95, respectively; S4D Figs. and not shown). These results show that our 384-well plate-based growth kinetics assay produces highly reproducible growth data that are in good agreement with previously published, lower throughput methods and suggest its suitability for high-throughput yeast trait profiling.

**Fig 4:**
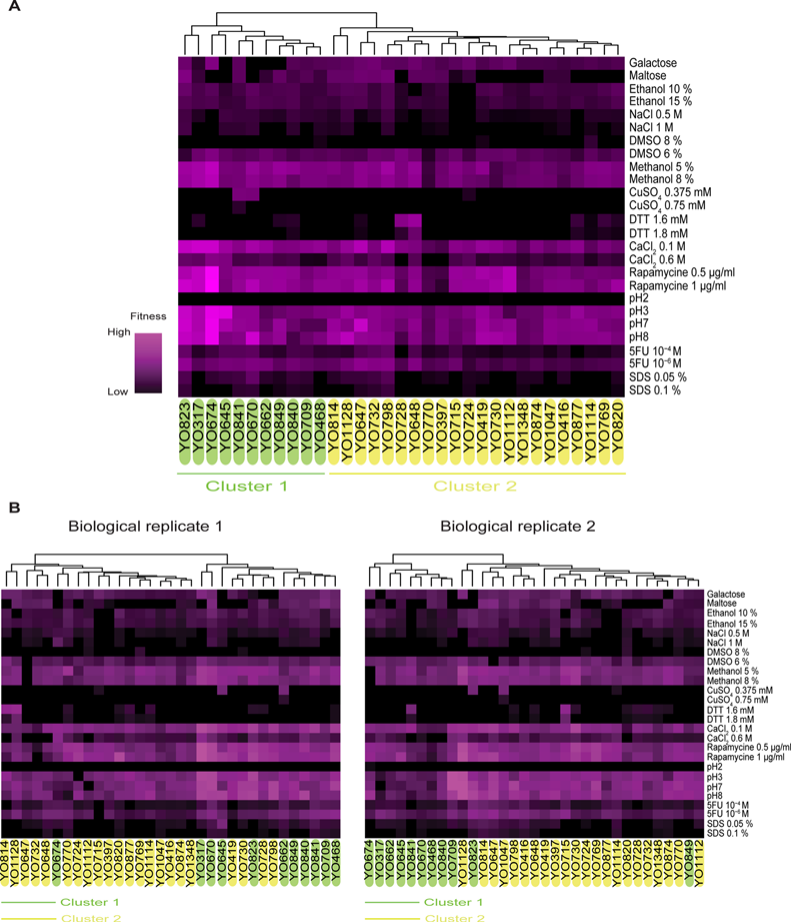
Trait profile analysis in a collection of 33 natural variants of *S*. *cerevisiae* Using microcultivations in 384-well plates with OD monitoring in a microplate reader, growth rates were determined for 33 natural *S*. *cerevisiae* isolates in the 26 indicated environmental conditions. The fitness is represented for each strain as the ratio between the growth rate in each of the 26 indicated conditions and the growth rate in a reference condition (minimal YNB medium containing 2% glucose). Violet squares depict rapid growth whereas black squares represent slow growth compared to growth in the reference condition. Hierarchical clustering was performed using a centred Pearson correlation metric and average linkage mapping using 2 biological replicates (A). Two main clusters are highlighted in green and yellow. The heat maps obtained when taking into account each biological replicate separately are shown in (B) and strains were coloured according to the clusters obtained for the averaged data in panel A.

To verify this assumption, we subjected a collection of 33 natural *S*. *cerevisiae* variants (S1 Table) to our growth assay in 26 environmental conditions, differing by type of carbon source, metal, toxin, or other compounds added or by pH (S1 Table). Hierarchical clustering of the measured growth rates (means of two independent experiments) revealed two main clusters (Fig. 4A). Although our sample set was likely too small (33 strains), too diverse (21 different ecological origins and 20 geographical origins), and the number of conditions tested too low to identify clear correlations between genetic background and ecological or geographical origin, it is relevant for this study to notice that similar clusters were found when analysing both experimental (biological) replicates separately (Fig. 4B). It should be noted that using our 384-well plate microcultivation assay, growth data generation across this set of 33 natural strains and 26 conditions took less than 3 weeks, whereas it would have taken about 3 months using 96-well plates (comparison based on the use of a single plate reader). Finally, the culture volume used here was 80 μl per well, whereas it usually ranges from 150–300 μl per well for the 96- or 100-well formats. This relatively small-scale trait profiling experiment suggests that our growth assay is reproducible enough for growth phenotyping screens in yeast and illustrates the considerably higher time- and cost-efficiency of this assay compared to existing methods.

### Application of the Growth Assay to Chronological Life Span Analysis

**Fig 5:**
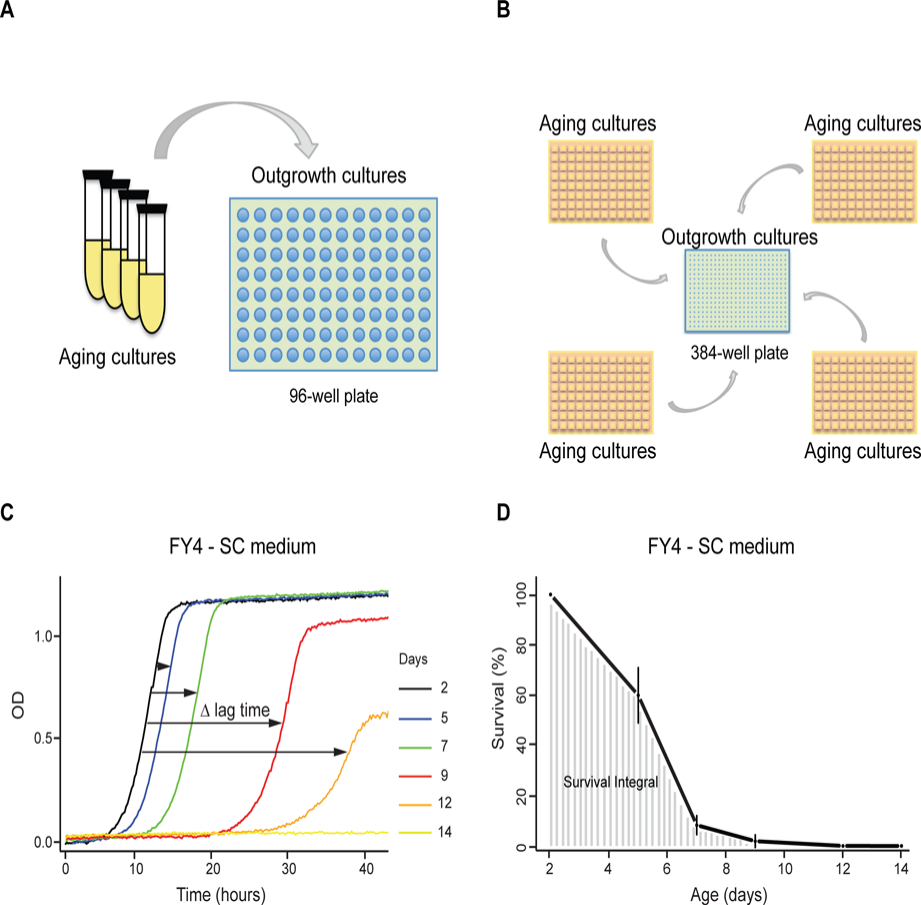
Comparison of outgrowth kinetics-based experimental pipelines for Chronological Life Span analysis. A) A previously described method [31] is based on aging cultures in tubes or flasks followed by outgrowth assays in 96-well plates whereas our approach (B) uses aging cultures maintained in 96-deep-well plates followed by outgrowth assays in 384-well plates. Both approaches rely on the analysis of high-density growth curves (doubling time and lag time) generated on successive days by inoculating fresh medium with a sample of the aging cultures (C) to eventually plot survival curves and determine survival integrals (D) as shown in this figure for the FY4 strain maintained in SC medium. The survival percentages shown in panel D are means ± SDs for 3 biological replicates.

**Fig 6:**
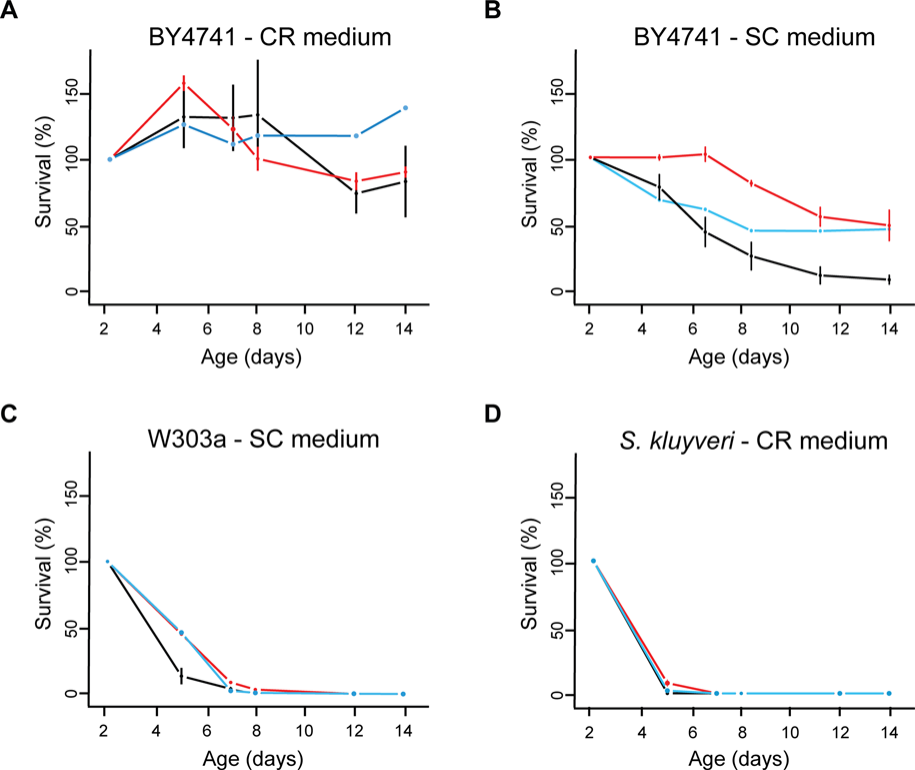
Chronological life span studies using different methods and yeast strains in rich or caloric restriction media. Survival curves were obtained by Colony Forming Unit counting using aging cultures in flasks (blue lines) or by kinetic outgrowth assays (in 384-well plates) maintaining aging cultures in 96-deep-well plates (black lines) or flasks (red lines). Chronological aging was estimated for the BY4741 and W303a *S*. *cerevisiae* strains and the *S*. *kluyveri* reference strain CBS3082 in synthetic complete medium with 0.5% glucose (CR medium) or 2% glucose (SC medium). The survival percentages shown are means ± SDs for 3 biological replicates.

Another phenotypic trait that can be investigated using our kinetic growth assay is the Chronological Life Span (CLS). Murakami and colleagues [31] described a method that allows quantification of chronological aging in yeast with a higher throughput than the commonly used assay based on counting Colony Forming Units (CFUs). Whereas for the latter assay, serial dilutions of samples taken from the aging cultures on successive days are plated on solid media, the former method involves inoculation of aliquots of the aging cultures into 100-well microplates containing fresh liquid medium, followed by OD monitoring for growth curve generation (Fig. 5A). One remaining limitation in this outgrowth kinetics-based approach is the number of aging cultures in flasks or in tubes that can be maintained in parallel during the CLS experiment. We aimed at overcoming this limitation and further increase the throughput of this CLS assay by (1) maintaining the aging cultures in 96-deep-well plates and (2) generating the outgrowth curves using the above described growth assay in 384-well plates (Fig. 5B). As described by Murakami et al. [31], the increase in lag time for outgrowth curves generated from the same aging culture on successive days (Fig. 5C) allows to quantify the decrease in the number of surviving cells remaining in the aging culture over the duration of the experiment (Fig. 5D). The survival curves in Fig. 6, obtained with different yeast strains in rich or caloric restriction medium, show a good agreement for viability estimations obtained by CFU counting or using the outgrowth assay (in 384-well format) after sampling from the same aging cultures maintained in flasks. Similar results were also obtained independently of whether the outgrowth assay was performed from aging cultures maintained in flasks or 96-deep-well plates (Fig. 6), although a higher discrepancy was observed for the BY4741 strain in SC medium. These results confirm that the combination of aging cultures maintained in 96-deep-well plates with outgrowth assays in 384-well plates is suitable for quantitative CLS analysis in yeast.

As outlined above, we developed a computer program (CLS analysis tool or CATHODE) that can be used in combination with the growth analysis tool GATHODE to accelerate data analysis of our higher throughput CLS assay. The outgrowth curves generated during a CLS experiment are first analysed in GATHODE. The. gat analysis output files linked to an aging experiment can then be opened in CATHODE to generate survival curves and calculate survival integrals for the tested strains and conditions (S5 Fig.). In order to provide a useful and simple microbial growth analysis tool to a larger community, we decided to keep GATHODE on a more generic level and to develop a separate GUI for CATHODE. Similar to GATHODE, CATHODE allows for blacklisting of certain wells and results can be exported as. csv tables for further analysis (S5B Fig.).

**Fig 7:**
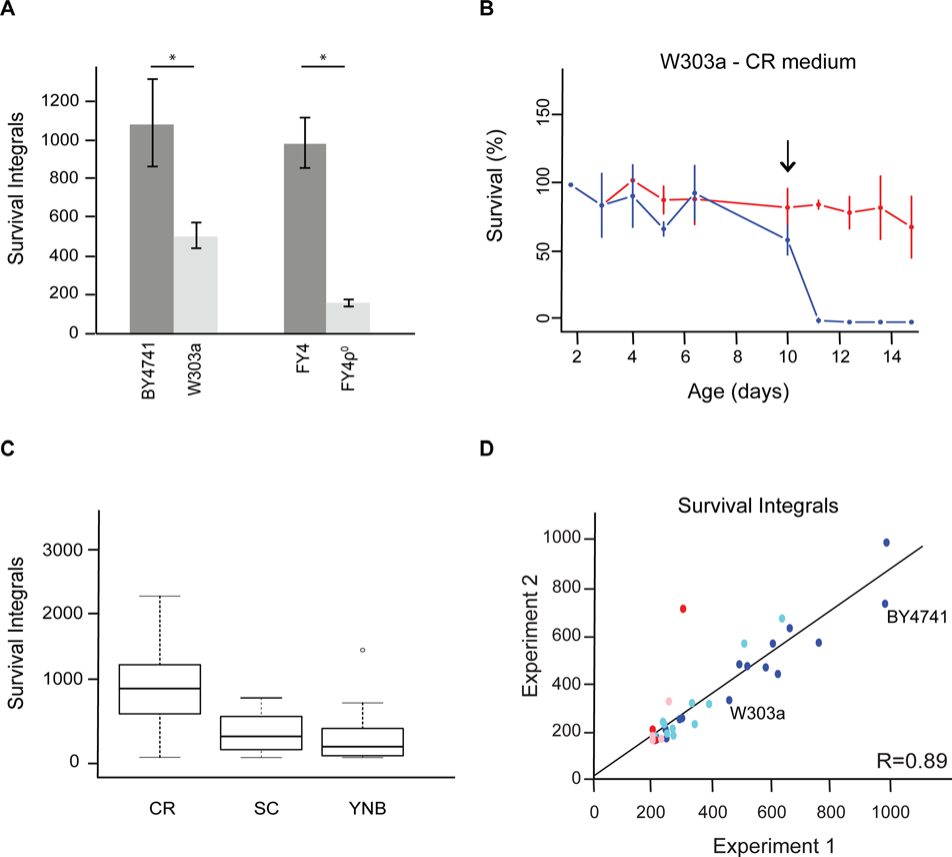
The developed strategy for CLS determination is reproducible and confirms previously reported features of yeast aging. A) Comparison of CLS of different *S*. *cerevisiae* strains maintained in SC medium (BY4741 and W303a strains) or CR (caloric restriction) medium (FY4 and FY4ρ^°^ strains). FY4ρ^°^ is a strain lacking mitochondrial genome. Unpaired t-tests on the average of 3 biological replicates indicate significant differences in chronological life span between these strains (*, p < 0.05). B) Survival curves for 3 biological replicates of the W303a strain in a CR medium without (red line) and with (blue line) 100 mM acetic acid added at day 10, as indicated by the arrow. C) Comparison of CLS for yeast cultures aged in CR medium (0.5% glucose) or media supplemented with 2% glucose (SC and YNB). Boxplots are shown for CLS values obtained with 41 different *S*. *cerevisiae* strains and 4 strains from other yeast species (*K*. *lactis*, *S*. *kluyveri*, *S*. *exiguus* and *Z*. *rouxii*) in the indicated growth media. D) Survival integrals for 16 *S*. *cerevisiae* strains obtained in two independent experiments in complete SC (dark blue dots) and minimal YNB (light blue dots) media. Survival integrals were also determined for other yeast species in these same SC (red dots) and YNB (pink dots) media. The values obtained for the BY4741 and W303a *S*. *cerevisiae* lab strains in SC medium are indicated by annotation.

To further validate our CLS assay, we tested whether it would reproduce properties of CLS in yeast that have previously been reported by others. Using our protocol, we found a shorter CLS for the W303a strain than for the BY4741 strain (Fig. 7A), as previously described for these two laboratory strains [23]. The lack of mitochondrial genome in *S*. *cerevisiae* (ρ^°^) has been shown to extend the RLS, but decrease the CLS [43, 44]. We determined the CLS for the laboratory strain FY4 and its isogenic FY4ρ^°^ counterpart lacking the mitochondrial genome and hence mitochondrial functions. The latter displayed a shorter survival than the FY4 strain, confirming the importance of mitochondrial integrity to support a long chronological life span (Fig. 7A). Moreover, also in agreement with previous studies, we found that the addition of extracellular acetic acid drastically decreases the proportion of surviving cells over time as determined here for the W303a strain in a caloric restriction (CR) medium (Fig. 7B) [45]. This medium is supplemented with only 0.5% glucose and usually increases the longevity of yeasts significantly compared to complete or minimal media containing 2% glucose [46]. Survival integrals determined with our method for different laboratory strains (BY4741, FY4, W303 in both haploid and diploid backgrounds) as well as for various mutants, confirm that the CLS is higher in CR medium (0.5% glucose) compared to complete SC or minimal YNB media (both supplemented with 2% glucose) (Fig. 7C).

To evaluate the reproducibility of our CLS assay, we determined the survival integrals for 20 different yeast strains in both SC and YNB media twice, in two independent experiments. The analysed strains comprised different laboratory strains and natural isolates of *S*. *cerevisiae*, as well as strains from other yeast species including *S*. *kluyveri*, *K*. *lactis* and *Z*. *rouxii*. Comparison of the survival integrals obtained in these two experiments showed a good correlation (Fig. 7D), confirming that our method is reproducible and suitable for investigating the mechanisms underlying the regulation of CLS in high-throughput studies. Interestingly, survival integral values varied considerably from one *S*. *cerevisiae* strain to another (Fig. 7D, dark and light blue dots). So far, mostly the reference S288c strain or the isogenic BY4741 strain have been used to study chronological aging in the budding yeast. A recent study highlights, however, that the S288c strain is a phenotypically extreme isolate that is not representative of the *S*. *cerevisiae* population [11]. These observations suggest that it will be important to extend aging studies to natural variants of the *S*. *cerevisiae* species to further exploit this model organism to discover new genes and/or pathways involved in the control of aging.

Interestingly, almost all of the other yeast species tested here exhibited a shorter CLS than *S*. *cerevisiae* in the same growth conditions (Fig. 6D; red and pink dots in Fig. 7D). Among them, the protoploid species *S*. *kluyveri* is of particular interest as it should be an ideal candidate to evaluate the impact of Whole Genome Duplication on aging. Indeed, *S*. *kluyveri* diverged from the *S*. *cerevisiae* lineage before the Whole Genome Duplication event took place during the evolutionary history of the Hemiascomycetes species [47]. *S*. *kluyveri* variants have been isolated from different ecological niches (soil, trees or insect guts) and geographical origins (North America, Asia, Europe) [48, 49]. This yeast species has a sexual cycle, meaning that it can live in both haploid and diploid states, which is a prerequisite for linkage mapping. CLS analysis, and more particularly QTL-type studies, in this collection of *S*. *kluyveri* variants could therefore also reveal new pathways involved in life span determination. This will be greatly facilitated by an already existing collection of *S*. *kluyveri* variants for which genome sequencing data are available [49] and by the high-throughput CLS protocol and the software tools that have been developed here.

## Conclusion

Here we have set up and validated protocols for accelerated fitness trait profiling and chronological aging studies in yeast. The methods are based on microcultivations in 384-well plates using a thermostated and shaking microplate reader. The main advantages of these approaches are ease of implementation in standard laboratory settings with highly increased throughput for both growth and aging studies in *S*. *cerevisiae*. Moreover, our approach can be extended to other yeast species (as shown in this study) and even other microorganisms and allows for reduction of cell culture volume. The latter feature leads to decreased experimental costs, most particularly when precious reagents need to be tested as for example in drug screens. We also developed two user-friendly, free and open source software packages, GATHODE and CATHODE, to greatly facilitate analysis and interpretation of the wealth of data that can be generated by our high-throughput growth and CLS assays, respectively. These programs are compatible with different microplate formats and can easily be installed on all major operating systems.

Using our CLS assay, we found a large heterogeneity of chronological life spans in a subset of 12 natural *S*. *cerevisiae* variants and even between closely related lab strains such as FY4 and W303. The use of an entire population of strains should thus open new windows for deciphering the genetic and molecular basis of the aging process as well as enhance our understanding of the evolutionary mechanisms underlying the control of life span. Aging QTL studies using the progeny of crosses between *S*. *cerevisiae* isolates with differing CLS behaviours have been designed based on the described protocols and programs to progress towards these aims.

## Supporting Information

S1 Fig Correction strategy for the non-linearity of OD measurements in high-density cultures. (A) Exponentially growing cultures (OD = 0.6–0.7; measured in a standard cuvette in a conventional spectrophotometer) of different *S*. *cerevisiae* strains were concentrated 10-fold (OD = 6–7) and 28 serial dilutions were prepared (1.1- to 100-fold). Cultures of the FY4 and FY5 strains were also harvested during stationary phase (FY4_stat and FY5_stat), and similarly concentrated and serially diluted. Dot plots of the OD calculated based on the dilution factors (OD calculated) versus the OD determined for the diluted cultures (OD measured) in a microplate reader revealed a non-linear curve at high cell-density as expected given the saturation limit of the optical detector of the plate reader. The fitted third-order polynomial curve is defined by the equation: OD_corr_ = 4.7757(OD_meas_)– 0.445(OD_meas_)^2^ + 1.4078 (OD_meas_)^3^. The latter was used to correct the ODs measured throughout this study, except if indicated otherwise. (B) Comparison of corrected (black dots) versus non-corrected (grey dots) ODs measured in a microplate reader for the FY4 strain (YPD medium) after log-transformation shows that the exponential phase is only minimally affected by the correction. (C) Comparison of corrected versus non-corrected ODs measured in a microplate reader for the FY4 strain cultivated in YPD medium without (black dots) or with (grey dots) 7% NaCl to illustrate the large effect of OD correction on yield of biomass values. All the OD values shown were corrected for background absorbance.
(TIF)

S2 Fig Growth curves obtained for different yeast species under optimized microcultivation conditions. OD was monitored for 10 technical replicates of *S*. *kluyveri* (A) and *K*. *lactis* (B) liquid cultures (384-well plate) grown at low shaking speed in a microplate reader. The curves shown are corrected for background in ‘medium-only’ wells but not for non-linearity of OD measurements at high cell density.
(TIF)

S3 Fig Growth curves obtained by OD monitoring of FY4 strain microcultivations in a microplate reader (384-well plate) using YPD medium supplemented with the indicated concentrations of glucose. A diauxic shift was observed for none of the glucose concentrations tested.
(TIF)

S4 Fig Comparison of growth data obtained using different cultivation supports. Six *S*. *cerevisiae* strains were cultivated in 96- or 384-well plates in YPD medium containing different concentrations of NaCl (0–4%) to compare specific growth rate (A), lag time (B), and yield of biomass (C). Four *S*. *cerevisiae* strains as well as strains from other yeast species were cultivated in flasks or 384-well plates in YPD medium containing different concentrations of NaCl (0–4%) for growth rate determination (D). The results shown are means from 3 biological replicates.
(TIF)

S5 Fig CLS Analysis Tool (CATHODE) software. A) Screenshot of the CATHODE graphical user interface. The chart represents a survival curve for an aging wild-type *S*. *cerevisiae* culture. Survival percentages are means ± SDs for 3 biological replicates. A list of the analysed strains with corresponding replicates and culture conditions is generated automatically based on sample labelling. B) The extracted CLS parameters can be exported to a. csv file for further processing as averages with corresponding variances (as shown in this table) or as single values obtained for individual replicates.
(TIF)

S1 Table List of natural variants of Saccharomyces cerevisiae used in this study. (DOCX)

S2 Table Environmental conditions used for trait profile analysis in this study. (DOCX)

S3 Table Composition of the Synthetic Complete medium used in this study. (DOCX)
