# Supplementary figures and images for: Protocols and Programs for High-Throughput Growth and Aging Phenotyping in Yeast
Source: PLoS One. 2015 Mar 30;10(3):e0119807. doi: 10.1371/journal.pone.0119807 (PMC4379057; doi:10.1371/journal.pone.0119807)

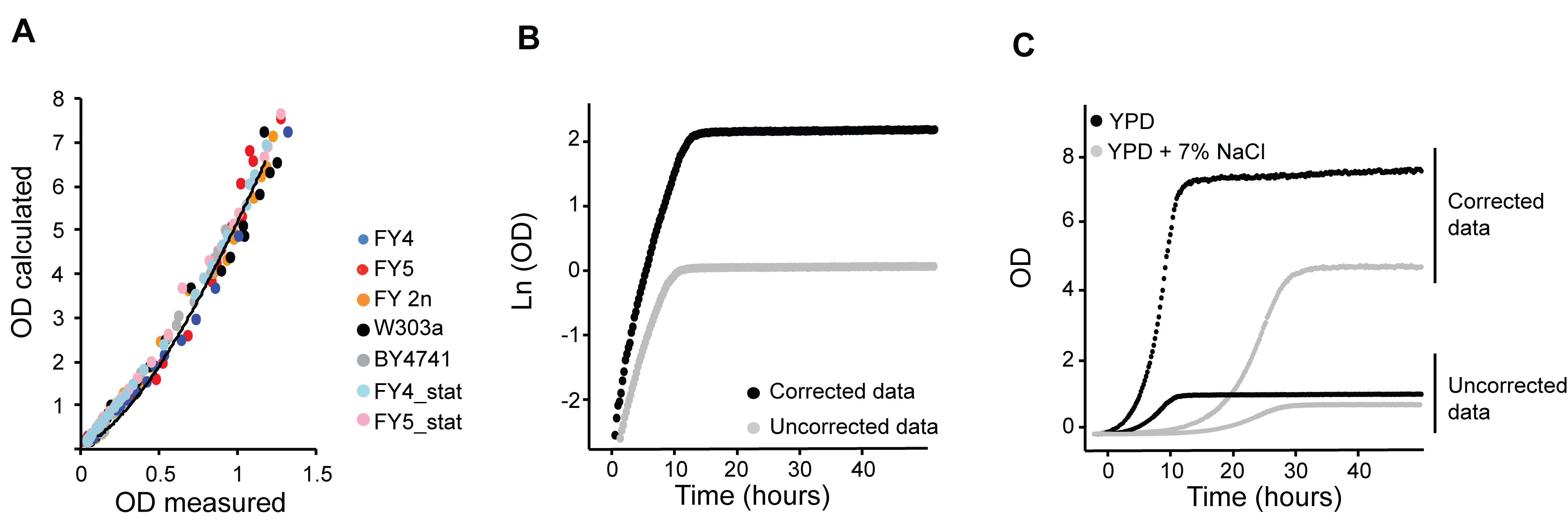

Supplement: S1 Fig — (A) Exponentially growing cultures (OD = 0.6–0.7; measured in a standard cuvette in a conventional spectrophotometer) of different S. cerevisiae strains were concentrated 10-fold (OD = 6–7) and 28 serial dilutions were prepared (1.1- to 100-fold). Cultures of the FY4 and FY5 strains were also harvested during stationary phase (FY4_stat and FY5_stat), and similarly concentrated and serially diluted. Dot plots of the OD calculated based on the dilution factors (OD calculated) versus the OD determined for the diluted cultures (OD measured) in a microplate reader revealed a non-linear curve at high cell-density as expected given the saturation limit of the optical detector of the plate reader. The fitted third-order polynomial curve is defined by the equation: ODcorr = 4.7757(ODmeas)– 0.445(ODmeas)2 + 1.4078 (ODmeas)3. The latter was used to correct the ODs measured throughout this study, except if indicated otherwise. (B) Comparison of corrected (black dots) versus non-corrected (grey dots) ODs measured in a microplate reader for the FY4 strain (YPD medium) after log-transformation shows that the exponential phase is only minimally affected by the correction. (C) Comparison of corrected versus non-corrected ODs measured in a microplate reader for the FY4 strain cultivated in YPD medium without (black dots) or with (grey dots) 7% NaCl to illustrate the large effect of OD correction on yield of biomass values. All the OD values shown were corrected for background absorbance. (TIF) [file pone.0119807.s001.tif]

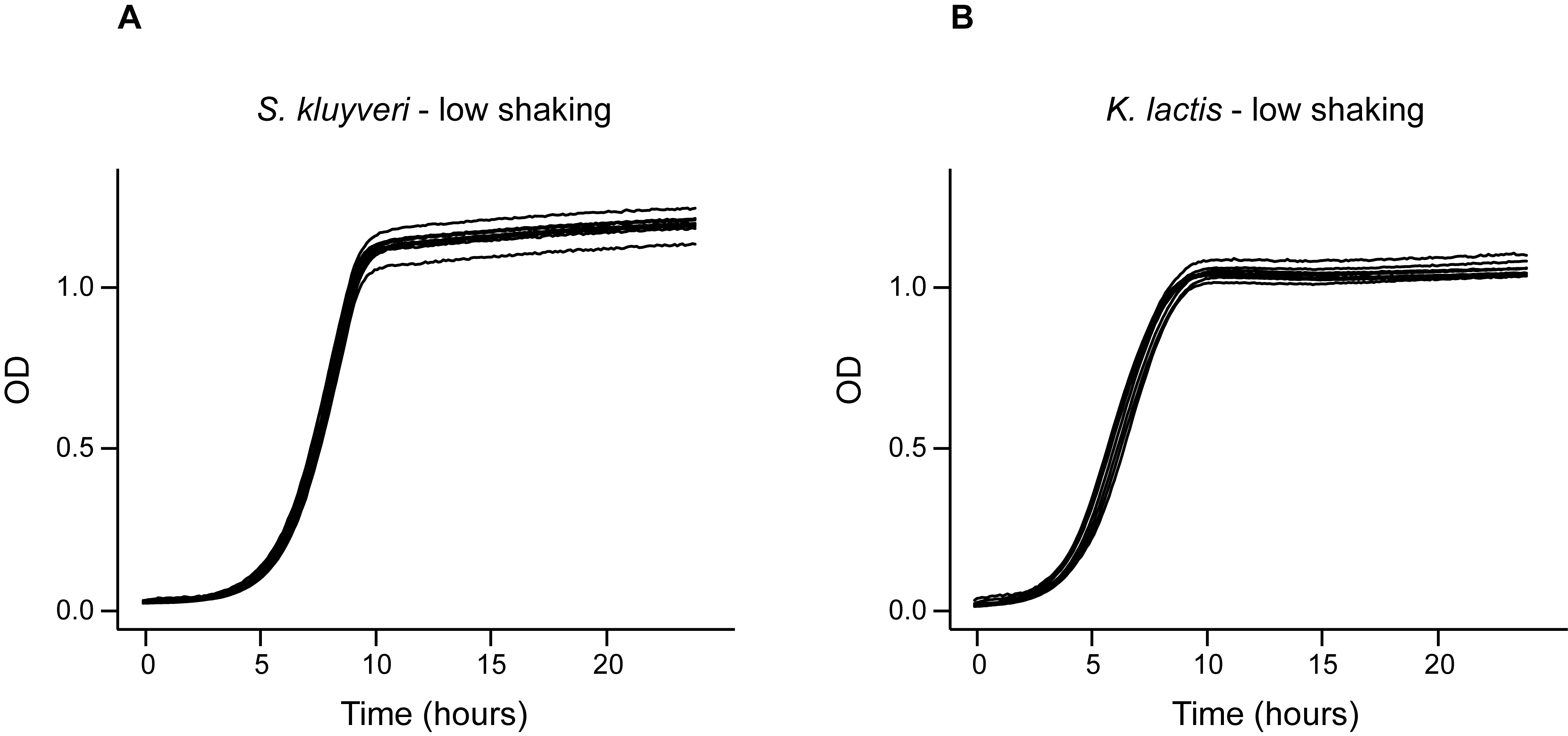

Supplement: S2 Fig — OD was monitored for 10 technical replicates of S. kluyveri (A) and K. lactis (B) liquid cultures (384-well plate) grown at low shaking speed in a microplate reader. The curves shown are corrected for background in ‘medium-only’ wells but not for non-linearity of OD measurements at high cell density. (TIF) [file pone.0119807.s002.tif]

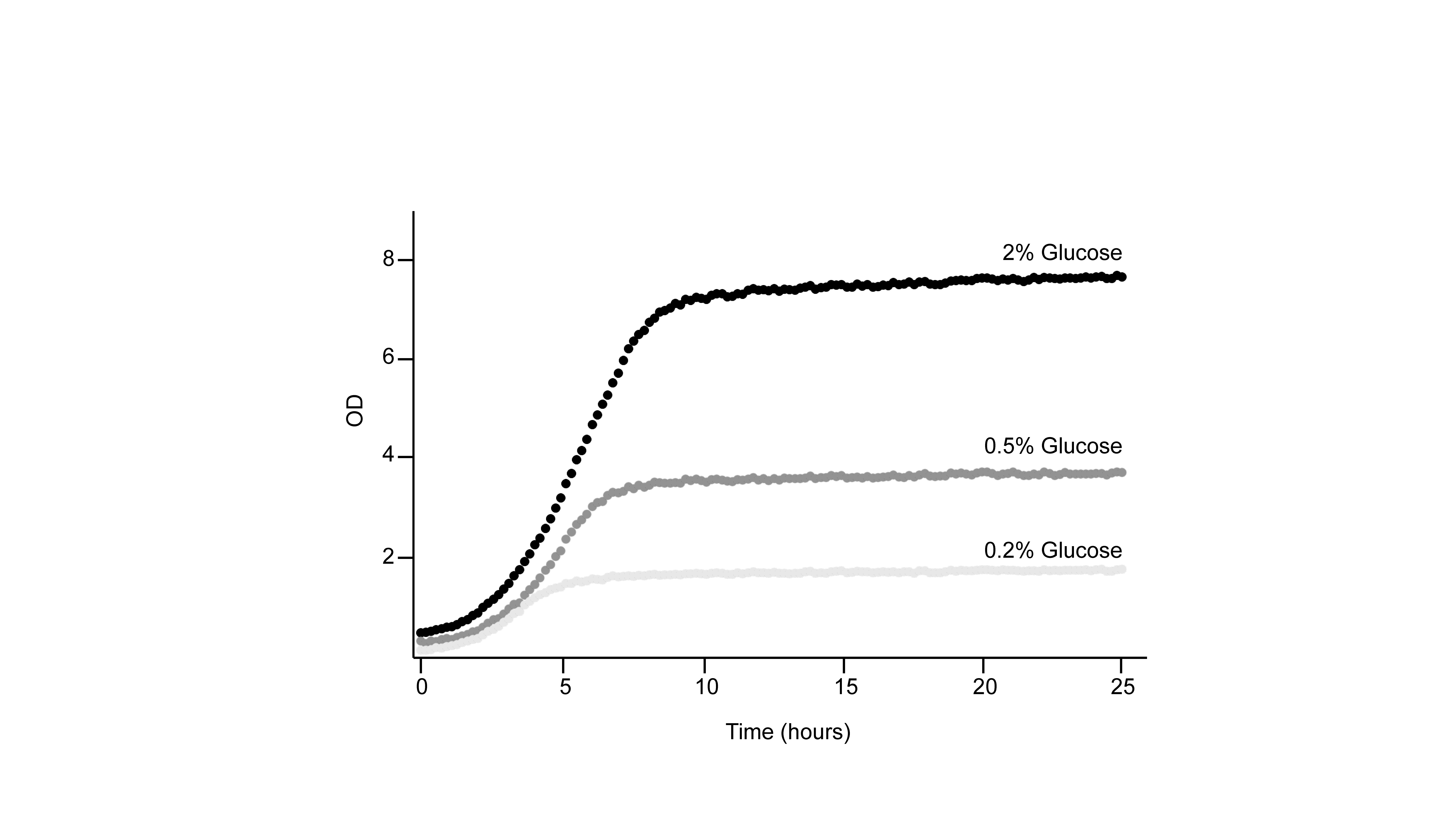

Supplement: S3 Fig — A diauxic shift was observed for none of the glucose concentrations tested. (TIF) [file pone.0119807.s003.tif]

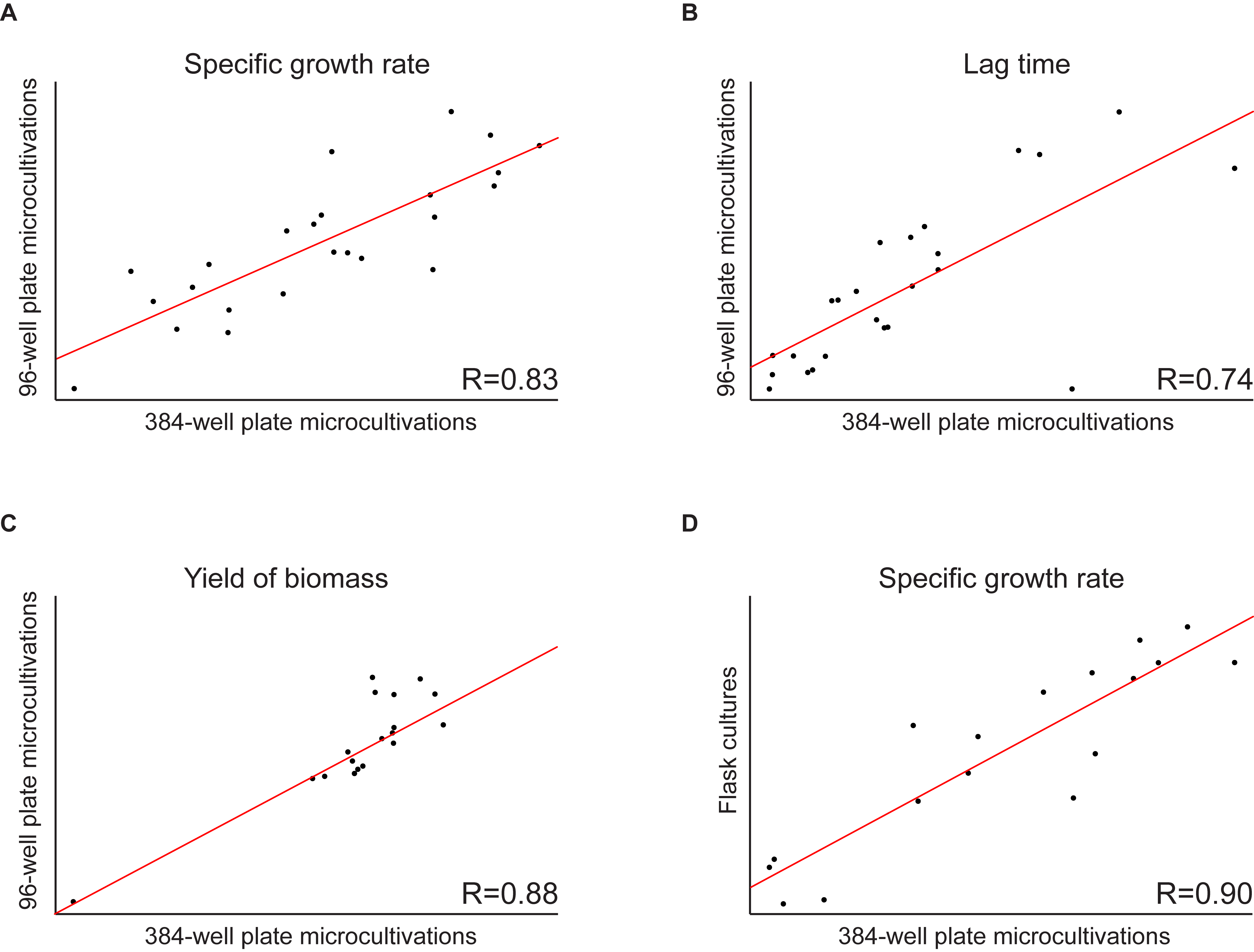

Supplement: S4 Fig — Six S. cerevisiae strains were cultivated in 96- or 384-well plates in YPD medium containing different concentrations of NaCl (0–4%) to compare specific growth rate (A), lag time (B), and yield of biomass (C). Four S. cerevisiae strains as well as strains from other yeast species were cultivated in flasks or 384-well plates in YPD medium containing different concentrations of NaCl (0–4%) for growth rate determination (D). The results shown are means from 3 biological replicates. (TIF) [file pone.0119807.s004.tif]

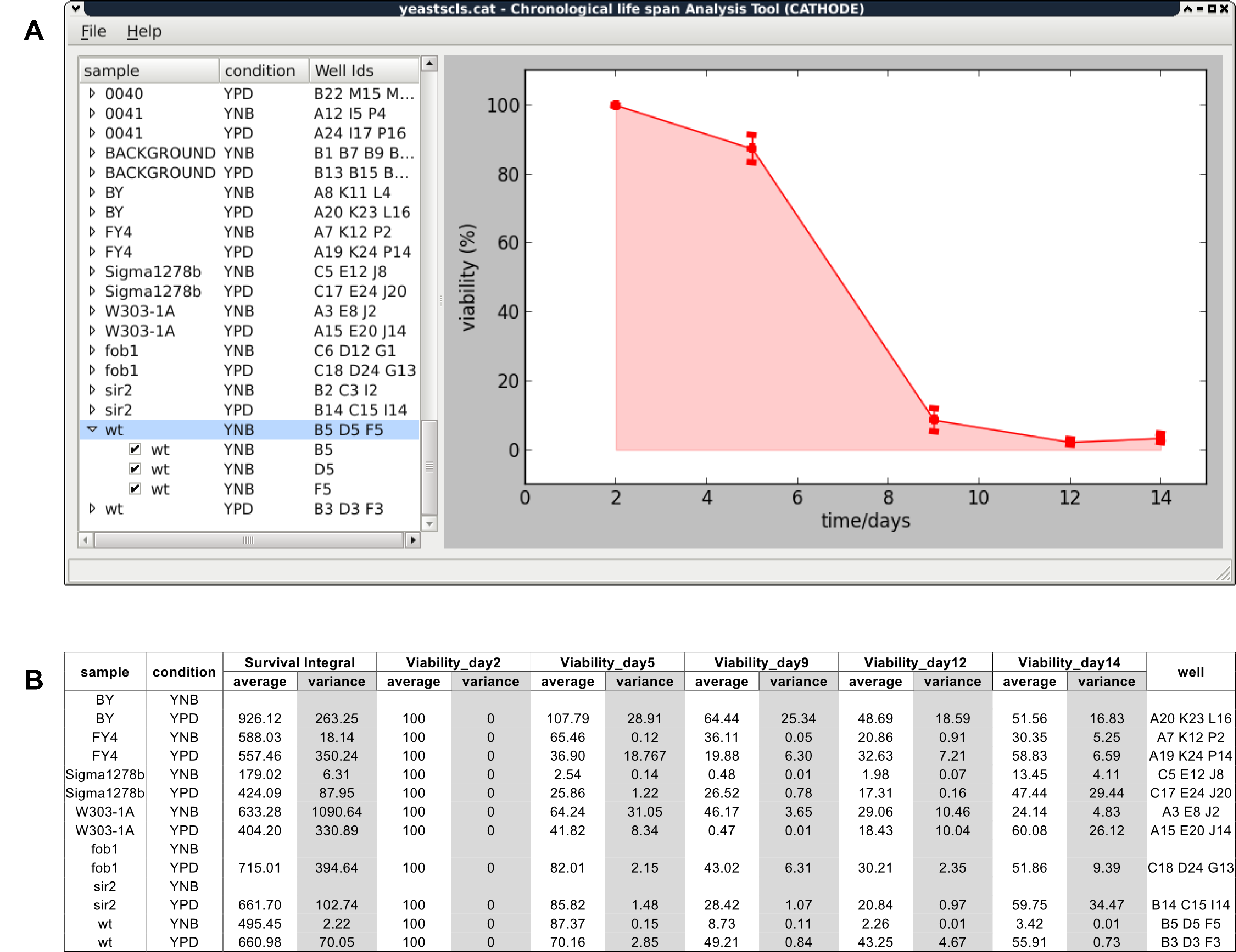

Supplement: S5 Fig — A) Screenshot of the CATHODE graphical user interface. The chart represents a survival curve for an aging wild-type S. cerevisiae culture. Survival percentages are means ± SDs for 3 biological replicates. A list of the analysed strains with corresponding replicates and culture conditions is generated automatically based on sample labelling. B) The extracted CLS parameters can be exported to a. csv file for further processing as averages with corresponding variances (as shown in this table) or as single values obtained for individual replicates. (TIF) [file pone.0119807.s005.tif]
