# Supplementary material for: Protocols and Programs for High-Throughput Growth and Aging Phenotyping in Yeast
Source: PLoS One. 2015 Mar 30;10(3):e0119807. doi: 10.1371/journal.pone.0119807 (PMC4379057; doi:10.1371/journal.pone.0119807)
Supplement: S1 Table — (DOCX) [file pone.0119807.s006.docx]

**S1 Table. List of natural variants of *Saccharomyces cerevisiae* used in this study.**

| Strain | Alias | Ecological origin | | Geographical origin | Assayed in this study for |
| --- | --- | --- | --- | --- | --- |
| YO317 | Y10 | | Coconut | Philippines | Growth |
| YO397 | UCD 05-780 | | Beetle from infested Prunus Bokhariensis | United States | Growth + CLS |
| YO416 | AY529517 | | Cacao fermentation | Ghana | Growth |
| YO419 | G89 | | Cacao fermentation | Ghana | Growth |
| YO468 | TY19 | | Togwa | Tanzania | Growth |
| YO645 | NRRLYB-210,  CBS428 | | Banana | Costa Rica | Growth |
| YO647 | NRRLY-12717 | | Murcha yeast cake | Nepal | Growth + CLS |
| YO648 | NRRLY-12769 | | Tapioca | Malaysia | Growth + CLS |
| YO662 | YB-427 | | Rum | Trinidad Tobago | Growth |
| YO670 | YB-3224 | | Wheat starch | United States | Growth |
| YO674 | YB-4085 | | Nip (palm) sap | Philippines | Growth |
| YO709 | YB-4082 | | Papaya | Philippines | Growth + CLS |
| YO715 | Y-1545 | | Stracchino cheese | Italy | Growth |
| YO724 | YB-4506 | | Bark from *Quercus variabi* (Asian Oak) | Unknown | Growth |
| YO728 | Y-11878,  ATCC26603 | | Cane juice | Jamaica | Growth + CLS |
| YO730 | YB-908 | | Wild cherry tree gum | Unknown | Growth + CLS |
| YO732 | Y-999 | | Amylo process | Unknown | Growth |
| YO769 | BF3 | | Mud, leaves | United States | Growth |
| YO770 | BF4 | | Mud, leaves | United States | Growth + CLS |
| YO798 | NRLLYB-1191 | | Citrus juice | United States | Growth |
| YO814 | NI1 | | Theo Chocolate cacao bean | Nigeria | Growth |
| YO820 | NG3 | | Theo Chocolate cacao bean | Nigeria | Growth |
| YO823 | NG6 | | Theo Chocolate cacao bean | Nigeria | Growth |
| YO840 | CR-IM35 | | Theo Chocolate cacao bean | Costa Rica | Growth |
| YO841 | CR-IM36 | | Theo Chocolate cacao bean | Costa Rica | Growth |
| YO849 | P-C2-CS2 | | Theo Chocolate cacao bean | Peru | Growth |
| YO874 | HAITI 11 | | Theo Chocolate cacao bean | Haiti | Growth |
| YO877 | MG2 | | Theo Chocolate cacao bean | Madagascar | Growth |
| YO1047 | Y-12638, CBS2888 | | Soil | South Africa | Growth + CLS |
| YO1112 | HC1-2 | | Green coffee beans | Honduras | Growth + CLS |
| YO1114 | MC1 1-1 | | Green coffee beans | Mexico | Growth + CLS |
| YO1128 | YE1-1 | | Green coffee beans | Yemen | Growth + CLS |
| YO1348 | GT2-3B | | Green coffee beans | Guatemala | Growth + CLS |

The strains have previously been described in Cromie et al., 2013 [33].
