# Supplementary material for: Protocols and Programs for High-Throughput Growth and Aging Phenotyping in Yeast
Source: PLoS One. 2015 Mar 30;10(3):e0119807. doi: 10.1371/journal.pone.0119807 (PMC4379057; doi:10.1371/journal.pone.0119807)
Supplement: S3 Table — (DOCX) [file pone.0119807.s008.docx]

**S2 Table. Environmental conditions used for trait profile analysis in this study.**

| Supplement | Concentration | Class |
| --- | --- | --- |
| Galactose | 2% | Carbon utilization |
| Maltose | 2% | Carbon utilization |
| 5-Fluorouracil | 0.001 – 0.1 mM | Environment & Metabolites |
| CaCl2 | 100 – 600 mM | Environment & Metabolites |
| CuSO4 | 0.375 – 0.750 mM | Environment & Metabolites |
| Ethanol | 10 – 15% | Environment & Metabolites |
| Methanol | 5 – 8% | Environment & Metabolites |
| NaCl | 500 – 1000 mM | Environment & Metabolites |
| pH8 |  | Environment & Metabolites |
| pH7 |  | Environment & Metabolites |
| pH3 |  | Environment & Metabolites |
| pH2 |  | Environment & Metabolites |
| DMSO | 4 – 8% | Toxins |
| DTT | 1.6 – 1.8 mM | Toxins |
| Rapamycine | 0.5 – 1μg/ml | Toxins |
| SDS | 0.05 – 0.1 % | Toxins |

The majority of growth assays were done in minimal YNB medium with 2% glucose. The classification “Carbon utilization” indicates that glucose was substituted by another carbon source. The pH was adjusted to values ranging from 2 to 8 using either NaOH or HCl. We used the same range of supplement concentrations and the same classification as in [11].
